# What lies beneath: a retrospective, population-based cohort study investigating clinical and resource-use characteristics of institutionalized older people in Catalonia

**DOI:** 10.1186/s12877-020-01587-8

**Published:** 2020-06-02

**Authors:** Jordi Amblàs-Novellas, Sebastià J. Santaeugènia, Emili Vela, Montse Clèries, Joan C. Contel

**Affiliations:** 1 Geriatric and Palliative Care Department, Hospital Universitari de la Santa Creu and Hospital Universitari de Vic, Barcelona, Spain; 2 Chair and Department of Palliative Care, University of Vic, Barcelona, Spain; 3 Central Catalonia Chronicity Research Group (C3RG), Centre for Health and Social Care Research (CESS), Universitat de Vic – University of Vic-Central University of Catalonia (UVIC-UCC), 08500 Vic, Spain; 4 Chronic Care Program, Ministry of Health, Generalitat de Catalunya, Travessera de les Corts, 131-159 08028 Barcelona, Catalonia Spain; 5 Unitat d’Informació i Coneixement, Servei Català de la Salut, Barcelona, Catalonia Spain

**Keywords:** Nursing home, Institutionalization, Multimorbidity, Use of resources, Older people

## Abstract

**Background:**

Planning population care in a specific health care setting requires deep knowledge of the clinical characteristics of the target care recipients, which tend to be country specific. Our area virtually lacks any descriptive, far-reaching publications about institutionalized older people (IOP). We aimed to investigate the demographic and clinical characteristics of institutionalized older people (IOP) ≥65 years old and compare them with those of the rest of the population of the same age.

**Methods:**

Retrospective analysis (total cohort approach) of clinical and resource-use characteristics of IOP and non-IOP older than 65 years in Catalonia (North-East Spain). Variables analysed included age and sex, diagnoses, morbidity burden—using Adjusted Morbidity Groups (GMA, *Grupos de Morbilidad Ajustada*)—, mortality, use of resources, and medications taken. All data were obtained from the administrative database of the local healthcare system.

**Results:**

This study included 93,038, 78,458, 68,545 and 67,456 IOP from 2011, 2013, 2015 and 2017, respectively. In this interval, an increase in median age (83 vs. 87 years), in women (68.64% vs. 72.11%) and in annual mortality (11.74% vs. 20.46%) was observed. Compared with non-IOP (*p* < 0.001 in all comparisons), IOP showed a higher annual mortality (20.46% vs. 3.13%), a larger number of chronic diseases (specially dementia: 46.47% vs. 4.58%), higher multimorbidity (15.2% vs. 4.2% with GMA of maximum complexity), and annual admissions to acute care (47.6% vs. 27.7%) and skilled nursing facilities (27.8% vs. 7.4%), mean length of hospital stay (10.0 vs. 7.2 days) and mean of medications taken (11.7 vs. 8.0).

**Conclusions:**

There is a growing gap between the clinical and demographic characteristics of age-matched IOP and non-IOP, which overlaps with a higher mortality rate of IOP. The profile of resources utilization of IOP compared with non-IOP strongly suggests a deficiency of preventive actions and stresses the need to rethink the care model for IOP from a social and health care perspective.

## Background

The persistently announced “epidemiological tsunami” of a great number of people of advanced age with multiple comorbidities, chronic conditions, and complex care needs is already a reality in our setting [1]. Besides the unquestionable impact on the quality of life of the people affected, organizational and/or economic repercussions of this “new reality” on health and social systems are also undeniable due to the use of resources and costs derived from care, as well as dependency- and disability-associated costs.

Beyond the fact that health systems need to keep promoting strategic measures to prevent or delay the occurrence of chronic diseases and disabilities [2], it is also essential to confront the reality of the most vulnerable people, who have high care needs and often reside in nursing homes. Based on the data available, during the first decade of the twenty-first century, there was a 150% increase in the number of nursing home beds, going from 37,281 in 2000 to 93,038 in 2011 in Catalonia [3]. This trend has been observed in most European countries, although it seems to have stabilized after 2011 [4].

Besides a higher global demand of nursing home beds, analysing the epidemiological and clinical characteristics of institutionalized people and assessing their needs, values and preferences are increasingly becoming central in the care model design for nursing homes. In 2013, Morley et al. already pointed out the need to research the care and characteristics of institutionalized people [5]. Since then, several analyses ―most of them led by Gordon’s group― have provided information about their health status, the effectiveness of health care models in care homes, and competencies and future challenges that should be faced in upcoming years [6]. International evidence suggest that the sociodemographic profile of institutionalized people has evolved in the last decades alongside the demographic shift [7, 8]. However, these trends cannot be confirmed in many countries that―like Spain―lack data from care facilities. Furthermore, the absence of quantitative data on the type and extent of resource utilization of residents admitted to care facilities in our area leaves policymakers with little options other than models from other countries (often heterogeneous in terminology and type of healthcare provided) to plan service provision in this setting.

Faced with this scenario, some authors have stressed the need to gather country-specific information about institutionalized patients to better understand the factors that influence mortality and morbidity and, therefore, the needs of these care models [9]. In response to this unmet need, “The Prevention and Attention to Chronicity Program” from Catalonia’s health system (northwest of Spain) has recently included an analysis of the characteristics and needs of institutionalized older people with three initial objectives: 1) describe the evolution—in the last 7 years—of epidemiological and clinical characteristics and the mortality of institutionalized older people (IOP) ≥65 years; 2) compare these characteristics with those of non-institutionalized older people (non-IOP) ≥65 years; and 3) compare the use of resources between IOP and non-IOP. Objectives 2) and 3) are expected to provide useful information for identifying and quantifying the differential needs of IOP compared to non-IOP.

## Methods

### Study design, participants, data source

This was a retrospective analysis of an administrative database that included all people ≥65 years in Catalonia (northwest of Spain) between 2011 and 2017. IOP were identified by using pharmaceutical dispensing coding data, which are necessary and specific to these people. Those subjects that in the year of inclusion had been living in a nursing home were considered IOP. Within the context of this study, and based on the consensus of the “*Integrated medical care model for older people residing in nursing homes”,* promoted by the local health authorities, the term “nursing home” was defined as any permanent or temporary place for people ≥65 years that do not have a sufficient degree of autonomy to perform daily activities, need constant supervision and live in a social-family situation requiring the replacement of their home.

Sociodemographic and clinical data were obtained from the Catalan Health Surveillance System (CHSS) that, since 2011, collects detailed information about the use of health care of the entire population of Catalonia. This record, which has been analysed in previous publications in other areas [10, 11], collects data about hospitalizations, primary care, specialized nursing care centres and mental health networks, information about prescriptions and pharmacy expenses, and a record of invoices, including outpatient visits, specialists, visits to the Emergency Room, non-urgent medical transportation, ambulatory rehabilitation, home oxygen therapy and dialysis. No data about hospital care in private health centres could be collected because these centres use different codes for patient identification.

### Variables

Age and sex were the demographic variables used in the study. Clinical variables included diagnoses, as they appear in the CHSS database based on the normal course of clinical practice, and coded according to the International Classification of Diseases, ninth revision, Clinical Modification (ICD-9-CM). The comorbidity burden was stratified based on the Adjusted Morbidity Groups (GMA, *Grupos de Morbilidad Ajustada*), which considers the type of disease—acute or chronic—, number of systems affected, and complexity of each disease, enabling to classify people in four strata based on their morbidity-associated risk: 1) Initial risk (healthy stage), with a GMA score up to the 50th percentile of the total population; 2) Low risk, with a GMA score between the 50th–80th percentiles; 3) Moderate risk, with a GMA score between the 80th–95th percentiles; and 4) High risk, with a GMA score above the 95th percentile [12, 13].

In order to describe the evolution of epidemiological and clinical characteristics and the mortality of IOP ≥65 years, a study of biannual, cumulative prevalence was conducted. The characteristics of IOP ≥65 years were compared with those of non-IOP in the same age group. The use of health resources between both groups for 2017 was also compared.

### Statistical methods

Categorical variables were described as numbers and percentages, whereas continuous variables were described as the mean and standard deviation (SD) and the median and interquartile range (IQR, defined by the 25th and 75th percentiles). Categorical variables were compared using the chi-square test. After confirming that all continuous variables followed a non-normal distribution (Kolmogorov-Smirnov test), we used the non-parametric Mann-Whitney U test for investigating between-group differences in these variables. The threshold of statistical significance was set at a bilateral alpha value of 0.05. All analyses were performed using the SPSS statistical software (IBM SPSS Statistics for Windows, Version 22.0. Armonk, NY: IBM Corp.).

## Results

### Evolution of epidemiological and clinical characteristics of older people institutionalized in nursing homes

During the seven-year interval analysed (2011–2017), the number of IOP tended to decrease, with a 27.5% reduction. IOP demographic, morbidity and mortality characteristics, summarized in Table 1, also changed during the analysed period. The prevalence of female subjects progressively increased by 3.5%, and the mean age and mortality increased by 3.9 years and 8.7%, respectively. Likewise, during the years under study, the prevalence of certain diseases in IOP increased, including heart failure (10.5%), COPD (3.0%), asthma (2.0%), chronic renal failure (14.7%), dementia (13.1%), depression (20.9%), stroke—cerebrovascular accident—(11.9%), chronic diseases of the musculoskeletal system (16.5%) and decubitus ulcers (3.3%). On the contrary, throughout the 7 years analysed, non-IOP demographic, morbidity and mortality characteristics did not undergo any relevant changes (Table 1).

**Table 1:** Comparative results of demographic, morbidity and mortality characteristics between institutionalized (IOP) and non-institutionalized older people (non-IOP). Years 2011, 2013, 2015 and 2017

		2011			2013			2015			2017		
		IOP	n-IOP	***p***	IOP	n-IOP	***p***	IOP	n-IOP	***p***	IOP	n-IOP	***p***
No. of cases		93,038	1,258,204	–	78,458	1,320,726		68,545	1,367,075		67,456	1,405,167	
Percentage		6.9	93.1	–	5.6	94.4	–	4.8	95.2	–	4.6	95.4	–
Age (years)	Mean (SD)	82.02 (8.4)	75.59 (7.6)	< 0.0001^a^	83.75 (8.0)	75.56 (7.7)	< 0.0001 ^a^	85.08 (7.8)	75.64 (7.8)	< 0.0001 ^a^	85.95 (7.6)	75.67 (7.8)	< 0.0001 ^a^
	Median (IQR)	83 (76–88)	75 (69–81)		85 (79–90)	76 (70–82)		86 (81–91)	74 (69–81)		87 (82–91)	74 (69–81)	
Sex	Men (%)	31.36	43.25	< 0.0001	29.29	43.52	< 0.0001	28.48	43.57	< 0.0001	27.89	43.71	< 0.0001
	Women (%)	68.64	56.75		70.91	56.48		71.52	56.43		72.11	56.29	
Deaths (%)		11.74	3.10	< 0.0001	14.1	3.40	< 0.0001	18.2	3.24	< 0.0001	20.46	3.13	< 0.0001
MORBIDITY													
Diabetes (%)		31.4	26.04	< 0.0001	29.68	24.75	< 0.0001	30.77	25.28	< 0.0001	32.15	25.75	< 0.0001
Heart failure (%)		18.2	9.38	< 0.0001	21.49	9.43	< 0.0001	25.8	10.26	< 0.0001	28.73	10.74	< 0.0001
COPD (%)		17.78	13.78	< 0.0001	18.00	13.27	< 0.0001	19.40	14.34	< 0.0001	20.77	15.14	< 0.0001
Hypertension (%)		84.59	74.11	< 0.0001	74.85	65.34	< 0.0001	78.08	66.30	< 0.0001	80.22	66.55	< 0.0001
Chronic kidney disease (%)		15.33	10.59	< 0.0001	18.99	10.86	< 0.0001	24.70	13.46	< 0.0001	29.98	15.99	< 0.0001
Asthma (%)		6.09	5.49	< 0.0001	6.55	5.89	< 0.0001	7.12	6.51	< 0.0001	8.11	7.09	< 0.0001
Dementia (%)		33.39	6.61	< 0.0001	35.82	4.55	< 0.0001	42.08	4.50	< 0.0001	46.47	4.58	< 0.0001
Cirrhosis (%)		1.43	1.41	0.5	1.18	1.11	0.459	1.31	1.3	0.878	1.43	1.43	0.998
Depression (%)		29.84	1793	< 0.0001	29.9	17.28	< 0.0001	33.69	19.13	< 0.0001	40.72	22.05	< 0.0001
Mental health chronic patient (%)		6.85	2.83	< 0.0001	10.07	3.25	< 0.0001	12.71	3.63	< 0.0001	15.36	4.28	< 0.0001
Stroke (%)		19.31	9.09	< 0.0001	22.78	8.87	< 0.0001	27.24	9.82	< 0.0001	31.16	10.77	< 0.0001
Ischemic heart disease (%)		21.66	15.37	< 0.0001	14.89	11.55	< 0.0001	15.65	11.86	< 0.0001	16.22	12.22	< 0.0001
Neoplasia (%)		19.99	19.65	0.012	19.54	18.40	< 0.0001	20.18	19.26	< 0.0001	22.66	21.42	< 0.0001
Chronic musculoskeletal diseases (%)		58.2	54.47	< 0.0001	61.62	55.54	< 0.0001	66.68	60.46	< 0.0001	74.73	67.22	< 0.0001
Osteoporosis (%)		19.33	16.04	< 0.0001	17.88	14.43	< 0.0001	20.59	15.54	< 0.0001	24.24	16.77	< 0.0001
Osteoarthritis (%)		43.64	37.03	< 0.0001	46.04	36.8	< 0.0001	50.36	40.26	< 0.0001	56.08	43.5	< 0.0001
Arthritis (%)		5.82	4.32	< 0.0001	6.09	4.33	< 0.0001	7.45	5.83	< 0.0001	9.41	7.55	< 0.0001
Chronic pain (%)		1.16	0.56	< 0.0001	1.46	0.84	< 0.0001	2.64	1.35	< 0.0001	4.41	1.9	< 0.0001
Malnutrition (%)		0.52	0.17	< 0.0001	0.79	0.25	< 0.0001	1.21	0.34	< 0.0001	1.55	0.41	< 0.0001
Pressure ulcer (%)		2.34	0.50	< 0.0001	3.40	0.59	< 0.0001	4.96	0.76	< 0.0001	5.67	0.86	< 0.0001

***COPD*** Chronic Obstructive Pulmonary Disease. ***IOP*** Institutionalized Older People. ***non-IOP*** Non-Institutionalized Older People. ***IQR*** interquartile range (25th and 75th percentiles). ***SD*** Standard deviation

^a^Both T-test and Mann-Whitney U test

**Fig. 1:**
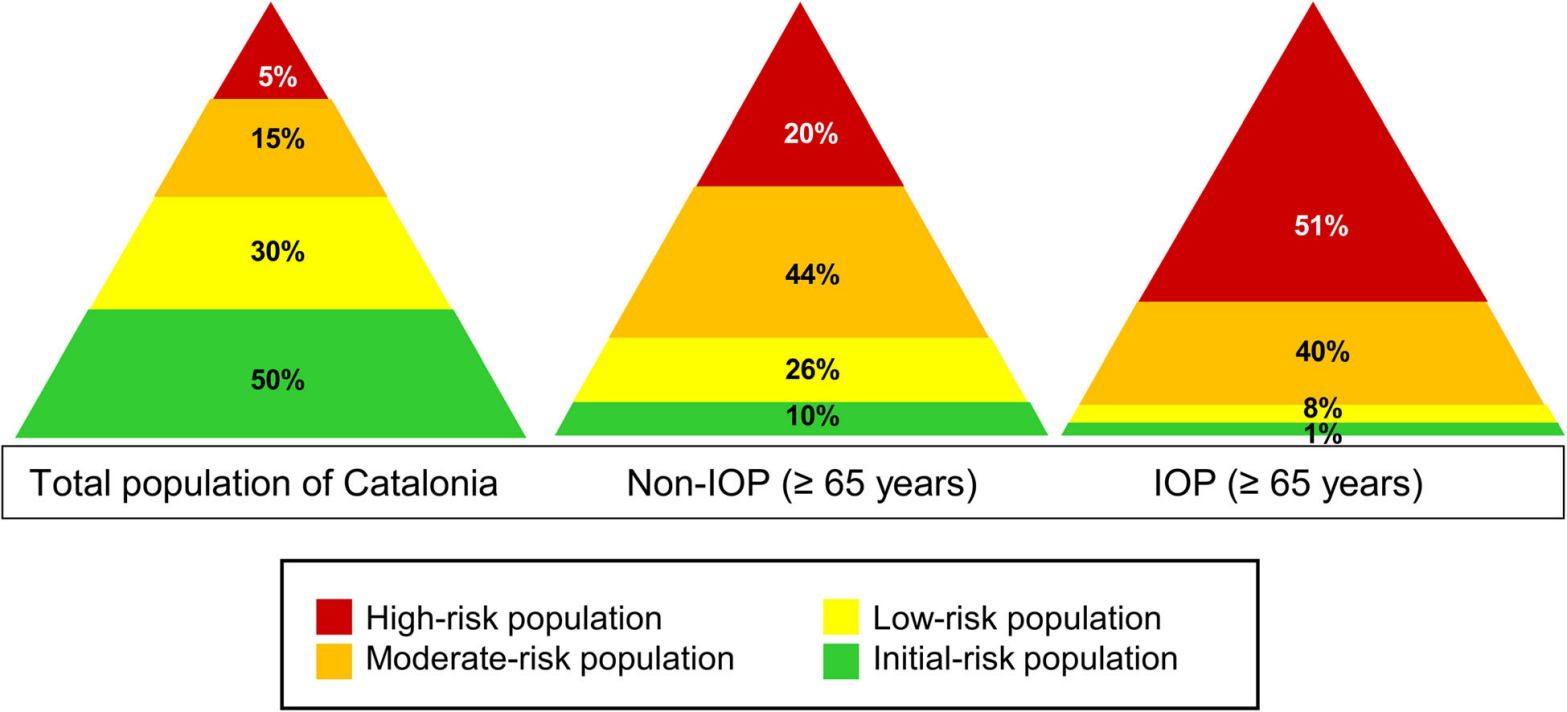
Comparative result of multimorbidity (GMA)-based stratification between the general population of Catalonia, non-institutionalized (non-IOP) ≥65 years and institutionalized older people ≥65 years (IOP) 2017

The analysis—narrowed down to 2017—showed age and sex distribution differences between IOP and non-IOP (Figure A1, Additional file 1), as well as differences in the prevalence of diseases and chronic conditions between both sexes, of which cardiac failure was the most prevalent with no statistically significant differences between women and men (Figure A2, Additional file 1). On the other hand, the analysis of the comorbidity burden using GMA narrowed down to 2017 showed differences in the proportion of IOP and non-IOP assigned to a certain risk, with 51% for the IOP high-risk population (Fig. 1).

### Comparative study of health resource use based on location

Regarding resource use by non-IOP and IOP (Table 2), significant differences were found in the annual percentage of acute care admissions (27.7% vs. 47.6%)—an even higher difference if only the percentage of urgent admissions is considered (13.6 vs 40.3%)—as well as in mean stay in these centres (7.2 vs. 10.0 days). There were also differences in the percentage of admissions to nursing skilled facilities (7.4% vs. 27.8%). On the contrary, the number of contacts with Primary Care teams had a difference of only 0.11 points between both groups. Regarding the medications taken, significant differences were detected in the number of medications taken and containers dispensed (Table 2).

**Table 2:** Comparative results of the use of resources between institutionalized and non-institutionalized men and women. Year 2017

HEALTH RESOURCE USE		IOP			non-IOP			
		Men	Women	Overall	Men	Women	Overall	***P***^**a**^
Visits to primary care	Mean (SD)	13.74 (15.5)	11.76 (13.3)	12.26 (14.0)	11.78 (13.7)	12.44 (11.7)	12.15 (11.7)	< 0.0001
	Median (IQR)	9 (4–18)	8 (3–16)	8 (3–16)	9 (4–15)	8 (4–16)	9 (4–16)	
Admissions to acute care hospitals (%)		63.03	41.58	47.56	32.13	24.19	27.66	< 0.0001
Urgent admissions to acute care hospitals (%)		52.31	35.72	40.34	16.45	11.41	13.61	< 0.0001
Patients with > 1 urgent admission to hospital (%)		11.79	7.05	3.55	3.30	2.05	2.34	< 0.0001
Length of hospital stay (days)	Mean (SD)	4.20 (9.9)	2.62 (7.3)	3.06 (8.1)	1.67 (7.0)	1.08 (5.2)	1.34 (6.1)	< 0.0001
	Median (IQR)	0 (0–4)	0 (0–1)	0 (0–1)	0 (0–0)	0 (0–0)	0 (0–0)	
Length of hospital stay (only acute care hospitals) (days)	Mean (SD)	11.24 (13 (4)	9.41 (11.2)	10.03 (12.1)	8.14 (13.7)	6.34 (11.2)	7.21 (12.5)	< 0.0001
	Median (IQR)	7 (3–15)	6 (2–12)	7 (2–13)	4 (0–10)	2 (0–8)	3 (0–9)	
Length of hospital stay for urgent admissions (days) (over all admissions)	Mean (SD)	3.73 (9.0)	2.40 (6.8)	2.77 (7.5)	1.25 (5.9)	0.83 (4.5)	1.01 (5.1)	< 0.0001
	Median (IQR)	0 (0–3)	0 (0–0)	0 (0–0)	0 (0–0)	0 (0–0)	0 (0–0)	
Length of hospital stay for urgent admissions (days) (over urgent admissions)	Mean (SD)	11.39 (12.6)	9.66 (10.7)	10.24 (11.4)	11.28 (14.0)	9.99 (12.2)	10.65 (13.2)	< 0.0001 | 0.286 ^b^
	Median (IQR)	8 (3–15)	7 (3–12)	7 (3–13)	7 (3–14)	7(3–12)	7(3–13)	
Admissions to emergency services	Mean (SD)	1.21 (1.7)	0.89 (1.4)	0.89 (1.5)	0.60 (1.3)	0.56 (1.2)	0.58 (1.3)	0.006 | < 0.0001 ^b^
	Median (IQR)	1 (0–2)	0 (0–1)	0 (0–1)	0 (0–1)	0 (0–1)	0 (0–1)	
Visits to specialized hospital care	Mean (SD)	2.18 (3.8)	1.45 (2.9)	1.65 (3.2)	3.44 (5.1)	2.95 (4.5)	3.17 (4.7)	< 0.0001
	Median (IQR)	1 (0–3)	0 (0–2)	0 (0–2)	2 (0–5)	1 (0–4)	1 (0–4)	
Visits to outpatient mental health services (%)		14.22	8.99	10.45	4.99	9.43	7.48	< 0.0001
Admissions to nursing skilled facilities (%)		34.97	24.99	27.77	7.69	7.21	7.42	< 0.0001
Dispensed medicines (No. of packages)	Mean (SD)	93.98 (79.9)	90.37 (67.3)	91.38 (71.1)	56.52 (65.3)	56.15 (56.7)	56.31(60.6)	< 0.0001
	Median (IQR)	82 (49–122)	80 (47–119)	81 (47 (120)	43 (17–78)	44 (18–80)	44 (17–79)	
Prescribed medicines (different active ingredients)	Mean (SD)	12.07 (5.5)	11.60 (5.5)	11.73 (5.5)	7.63 (5.6)	8.24 (5.6)	7.97 (5.6)	< 0.0001
	Median (IQR)	12 (8–15)	11 (8–15)	11 (8–15)	7 (3–11)	8 (4–12)	7 (4–11)	

^a^ Categorical variables were compared using a ANOVA test, whereas continuous variables were compared using the U Mann-Whitney test. Unless otherwise specified, the *p*-value corresponds to both, man/woman and IOP/non-IOP comparisons

^b^*p*-values correspond to man/woman | IOP/non-IOP

***IOP*** Institutionalized Older People. ***non-IOP*** Non-Institutionalized Older People. ***IQR*** Interquartile range (25th and 75th percentiles). ***SD*** standard deviation

## Discussion

Data obtained in this observational, retrospective study, including all IOP ≥65 years or non-IOP from nursing homes of Catalonia during 2011–2017, show an increase in IOP mean age and women proportion. Despite observing a reduction in the total number of IOP in our region, these people show greater morbidity, mortality and resource use than non-IOP.

The lack of consensus to define the concept of “nursing home” [14] makes it difficult to compare results from different studies conducted at a national and international level. However, Spain’s official data indicate that the prevalence of IOP in the region where the study was conducted is notably higher than in the rest of the country: 5.9% vs. 3.7% of the total population ≥ 65 years, respectively [15]. Furthermore, although Spanish data indicate an increase of IOP in the first decade of the century, our data show a progressive reduction—from 7.4% in 2011 to 4.8% in 2017—, a fact that could be related with the economic crisis and the difficulty to afford a nursing home [16]. Regardless of the reasons that may explain this trend, during the second decade of the twenty-first century, nursing home occupancy is also in a standstill in other European countries [4].

Concurrently with the increased occupation, various authors have highlighted an ageing trend in residential populations, partially explained by the ageing of the overall population [7, 8, 17]. In our cohort, the median age of non-IOP experienced a modest increase throughout the study period; conversely, the median age of IOP significantly increased from 83 years in 2011 to 87 in 2017. Like age, the sex distribution among residential populations has shown an evolving pattern, which may depend on the type of residential setting [7]. In our area, the percentage of women was persistently higher among IOP than non-IOP; however, sex distribution among IOP was rather constant throughout the investigated period.

Another key element, and a constant in the health care systems of most high-income countries [6, 18] is the tendency to concentrate those people with higher multimorbidities in a nursing home setting, a fact that underlines the imperative need to review/update the health care approach to these centres [19]. Compared to the rest of the population ≥ 65 years, IOP showed a higher prevalence of most chronic diseases (seven times higher in the case of dementia) and a four-times higher annual mortality. In fact, during the period analysed, mortality and morbidity, which virtually remained constant in non-IOP, increased in IOP despite a 27.5% decrease in the total number of IOP. These observations are consistent with epidemiological studies conducted in our setting, which confirm that the prevalence of IOP in end-of-life transitions is above 50%, with 70% of cases suffering from advanced dementia [20, 21]. In line with previous reports [22, 23], the prevalence of some comorbidities (including dementia) among IOP showed an increasing trend throughout the investigated period, reinforcing the idea that multimorbidity―most particularly, dementia―is an intrinsic characteristic of IOP and will be increasingly common in the residential setting. As mentioned previously [23], to improve IOP care, it is necessary to develop integrated care proposals from social and health care perspectives [24, 25]. This was, in fact, one of the motivations to develop the new “*Integrated medical care model for institutionalized older people”* in our region, the objective of which is to improve the duration and continuity of care of these people.

Consistently with studies recently conducted in our setting [26], our results show that IOP virtually present three times more urgent acute care admissions than the rest of the population ≥ 65 years; furthermore, our analysis revealed that the mean hospital stay of these patients is twice that of the general population of the same age range. As it has been repeatedly described in the literature, these observations confirm a close relationship between institutionalization and use of resources [27, 28]. It is therefore unavoidable to open the debate about the suitability and benefits of these admissions for patients’ health [29, 30], which are considered appropriate based on classic criteria [26]. In this context, it would be useful to analyse IOP hospitalizations that could be potentially prevented to better improve care planning. Likewise, the medication burden dispensed to IOP is 50% higher than to non-IOP in the same age group. This fact is of special concern since it is estimated that about 40% of this prescribing is inappropriate or suboptimal [31], at the same time causing a significant number of adverse events, hospital admissions and mortality [32].

Despite being a population with high care needs, no relevant differences were observed between IOP and non-IOP concerning the number of contacts between them and primary care teams (an increase of 0.1 visits per year), which suggest lack of preventive actions by the latter. This fact might explain, at least partially, IOP higher use of resources in acute care. However, the great variability of care models in our setting makes it difficult to draw conclusions in this sense, so studies that specifically investigate the difference regarding preventive actions between IOP and non-IOP would be necessary [33].

The results of this study must be interpreted in the context of some methodological limitations. On the one hand, it is very likely that isolated diagnoses collected in the normal course of clinical practice (and therefore subject to heterogeneous criteria), as well as morbidity groupers, do not properly capture the seriousness of clinical processes, mainly in fragile patients with comorbidity. Deepening the knowledge of the severity degree and progression of the diseases described, as well as other chronic conditions (primarily geriatric syndromes and cognitive decline), would enable to give a more accurate clinical description of IOP. Given the descriptive and population approach of the study, comparative analyses have not considered the likely more heterogeneous clinical characteristics of non-IOP—from healthy adults to those in end-of-life transitions—compared with IOP. It would be interesting to analyse paired cases with IOP and non-IOP in the future, for example, in home care programs. Finally, being a large-scale, database-dependent epidemiological study, one key element of the person-centred care process could not be addressed [34], namely their values and preferences [16], which would require a qualitative methodological approach.

## Conclusions

Our analysis shows that older people institutionalized in nursing homes tend to be increasingly older and more complex than the rest of the population of the same age. This growing gap between the two groups translates into higher mortality of IOP, which in our area was four times higher than that of non-IOP. Furthermore, the higher use of resources by acute care (especially hospitalizations) and medications of IOP suggests a deficiency of preventive actions. Taken together, our findings stress the need to rethink the care model for IOP from a social and health care perspective.

## Supplementary information

**Additional file 1. Appendix.** Figures. A1 and A2, which show the age and sex distribution of IOP and non-IOP, and the frequency of comorbidities of IOP

## Data Availability

The datasets used and/or analysed during the current study are available from the corresponding author on reasonable request.

## Abbreviations

CHSS: Catalan health surveillance system
COPD: Chronic obstructive pulmonary disease
GMA: Adjusted morbidity groups (Spanish: *Grupos de Morbilidad Ajustada*)
IOP: Institutionalized older people.
SD: Standard deviation
