# Supplementary material for: What lies beneath: a retrospective, population-based cohort study investigating clinical and resource-use characteristics of institutionalized older people in Catalonia
Source: BMC Geriatr. 2020 Jun 2;20:187. doi: 10.1186/s12877-020-01587-8 (PMC7265641; doi:10.1186/s12877-020-01587-8)
Supplement: Supplementary file 1 — Additional file 1. Appendix. Figures. A1 and A2, which show the age and sex distribution of IOP and non-IOP, and the frequency of comorbidities of IOP [file 12877_2020_1587_MOESM1_ESM.pdf]

Appendix

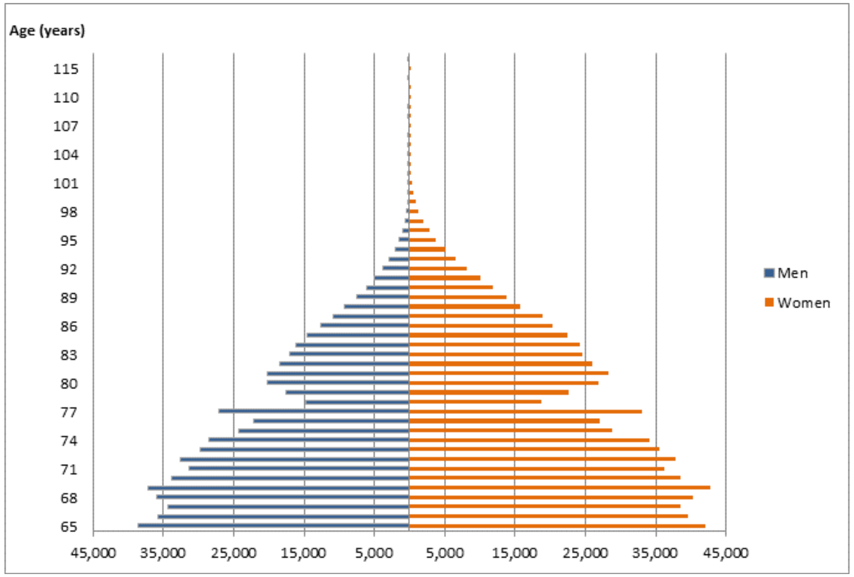

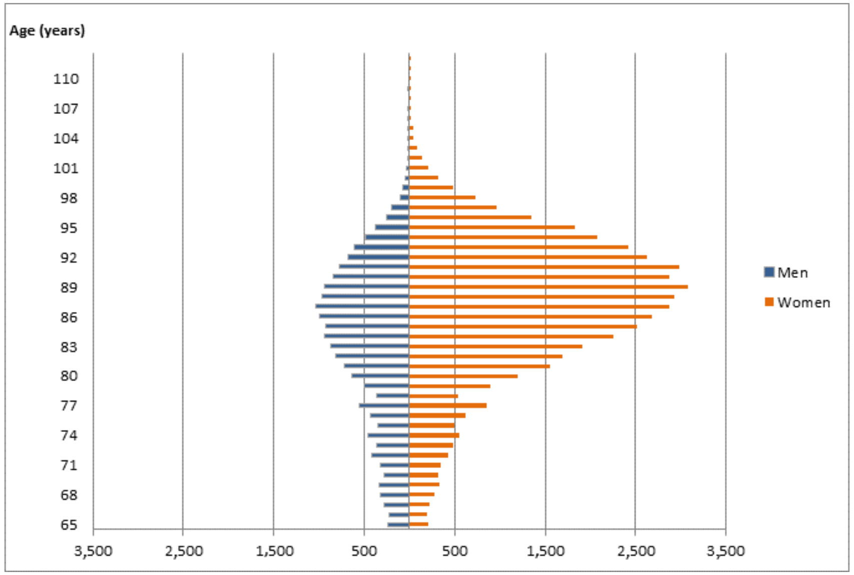

Fig. A1 Distribution by age and sex of non-IOP (A) and IOP (B), year 2017

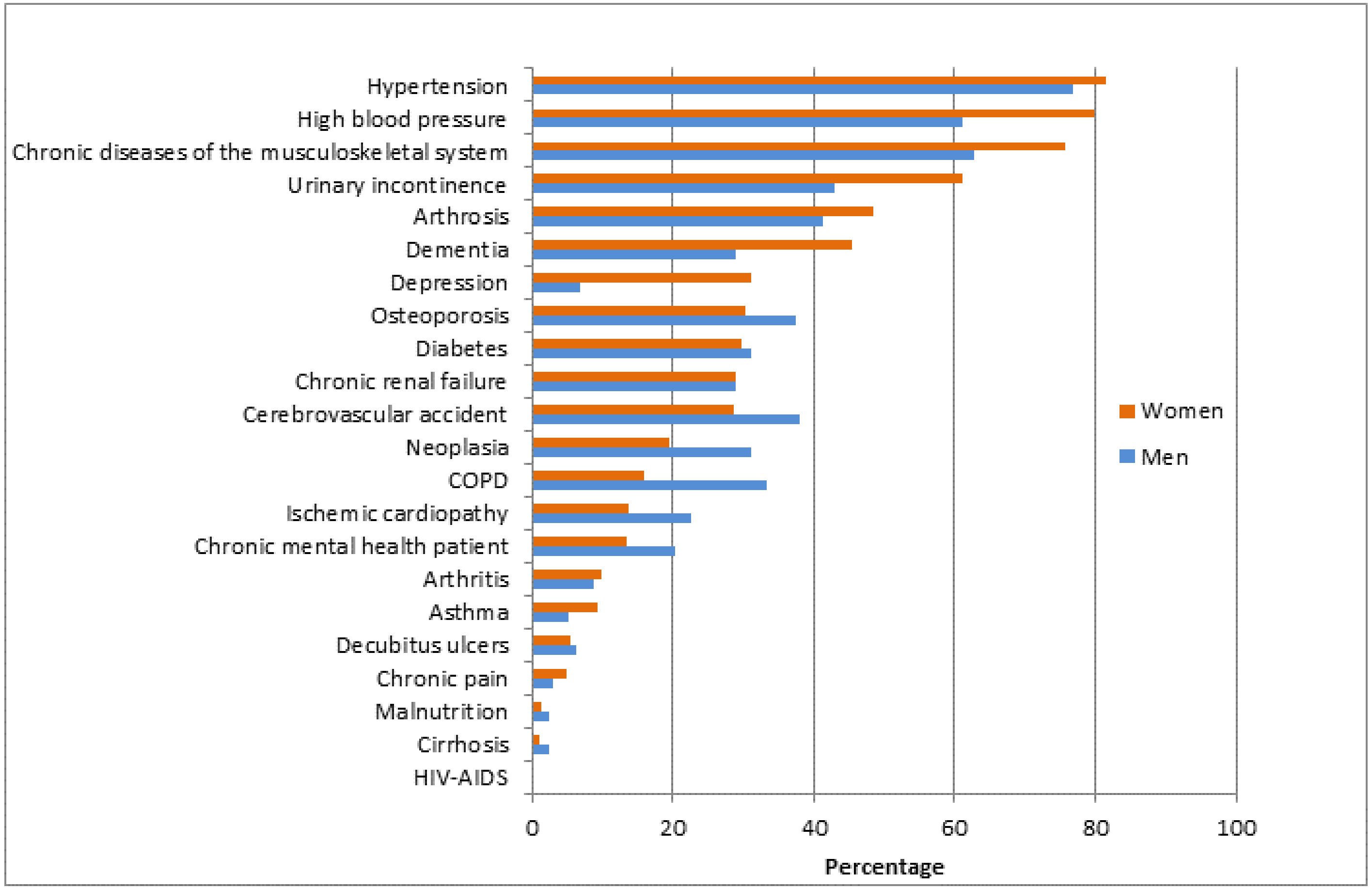

Figure A2: Morbidity of institutionalized patients by sex. Year 2017.
